# Inhibition of (p)ppGpp Synthesis and Membrane Fluidity Modulation by Diosgenin: A Strategy to Suppress *Staphylococcus aureus* Persister Cells

**DOI:** 10.3390/ijms26136335

**Published:** 2025-06-30

**Authors:** Yena Seo, Minjun Kim, Tae-Jong Kim

**Affiliations:** 1 Department of Forest Products and Biotechnology, Kookmin University, 77 Jeongneungro, Seongbukgu, Seoul 02707, Republic of Korea; yna127@kookmin.ac.kr (Y.S.); ykmj111@kookmin.ac.kr (M.K.); 2 Forest Carbon Graduate School, Kookmin University, 77 Jeongneungro, Seongbukgu, Seoul 02707, Republic of Korea

**Keywords:** diosgenin, persister cell, (p)ppGpp synthesis inhibition, *Staphylococcus aureus*, antibiotic tolerance, membrane potential modulation

## Abstract

Persister cells are a subset of bacterial cells that exhibit transient antibiotic tolerance without genetic resistance, contributing to the persistence of chronic infections. This study investigates the ability of diosgenin, a naturally occurring steroidal saponin, to inhibit persister cell formation in *Staphylococcus aureus* through metabolic suppression and membrane modulation. Diosgenin treatments at 80 µM and 160 µM significantly reduced persister cell survival under oxacillin, ciprofloxacin, and gentamicin stress, with reductions ranging from 82% to 94% after 3 h diosgenin pre-exposure. Gene expression analysis revealed that diosgenin downregulated *relP* and *relQ*, key genes involved in (p)ppGpp synthesis, by up to 60%, accompanied by 36–38% decreases in intracellular ATP levels. Diosgenin did not significantly alter membrane permeability or membrane potential but reduced membrane fluidity by 35% and 41% at 80 µM and 160 µM, respectively. Taken together, our findings suggest that diosgenin exerts a dual-action regulatory effect on persister cell formation by disrupting metabolic pathways essential for dormancy and altering membrane dynamics, potentially affecting membrane-associated signaling. This study provides a framework for the further exploration of diosgenin as a potential anti-persister agent with particular promise for use in combination with conventional antibiotics to enhance therapeutic efficacy against chronic bacterial infections.

## 1. Introduction

Bacterial persistence is a significant challenge in the treatment of chronic and recurrent infections caused by *Staphylococcus aureus* due to its ability to form persister cells [1]. Unlike genetically resistant strains, *S. aureus* cells exhibit transient antibiotic tolerance while in this metabolically dormant state, allowing them to evade antibiotic treatments without genetic mutation [2]. This reversible state is a critical survival strategy that enables persister cells to withstand bactericidal agents, breaking dormancy after antibiotic exposure, which contributes to infection relapse and prolonged treatment courses [3]. The clinical significance of persister cells is particularly pronounced in infections such as endocarditis, osteomyelitis, and cystic fibrosis, where repeated antibiotic treatment often fails to eradicate the entire bacterial population, resulting in persistent, refractory infections [1].

Existing therapeutic strategies for combating persister cells primarily focus on eradication through mechanisms that disrupt membrane integrity, interfere with metabolic processes, or induce oxidative stress [4,5]. Compounds like membrane-permeabilizing agents [6,7], DNA cross-linkers [8], and protease activators [9] have been investigated to target and eliminate preformed persister cells. However, these approaches have notable limitations. They generally fail to prevent the initial formation of persister cells and may induce collateral damage to non-persister bacterial populations or host tissues, thereby limiting their clinical applicability [10,11]. Moreover, most current strategies overlook the metabolic pathways involved in persister cell formation, focusing solely on killing dormant cells rather than preventing their development.

Addressing this gap, targeting the stringent response, a well-established regulatory pathway in bacterial persistence, has emerged as a promising approach [12]. The stringent response is mediated by the alarmone (p)ppGpp, a signaling molecule that downregulates cellular metabolism and promotes dormancy in response to nutrient deprivation, antibiotic stress, and other environmental cues [13]. In *S. aureus*, the synthesis of (p)ppGpp is regulated by the *relP* and *relQ* genes, both of which encode small (p)ppGpp synthases [14]. Previous studies have demonstrated that the genetic knockout of these genes significantly reduces persister cell formation, suggesting that inhibiting (p)ppGpp synthesis could effectively prevent persister cell establishment [15]. Despite this approach’s therapeutic potential, few compounds have been identified that specifically inhibit (p)ppGpp synthesis without inducing cytotoxic effects or compromising bacterial viability [16,17].

Natural compounds have recently gained attention as potential anti-persister agents due to their diverse pharmacological profiles and low toxicity compared to synthetic drugs [18,19,20]. Diosgenin, a naturally occurring steroidal saponin found in various plant species, is a notable example [21]. Structurally analogous to cholesterol, diosgenin can integrate into bacterial membranes, potentially affecting membrane fluidity, intracellular signaling, and metabolic pathways [22]. It has been extensively studied for its anticancer [23], anti-inflammatory [24], and anti-bacterial biofilm activities [25]. In the context of bacterial infections, diosgenin has been reported to inhibit biofilm formation in *S. aureus* by regulating sigma factor B (σ^B^), a key factor involved in stress response and biofilm development [26].

Despite its known biological functions, the details of diosgenin’s effects on persister cell formation and (p)ppGpp synthesis remain underexplored. Given its structural similarity to cholesterol, diosgenin may disrupt membrane potential and affect intracellular signaling pathways. Additionally, various physiological changes induced by diosgenin in bacteria suggest a potential impact on intracellular ATP levels; however, no direct evidence has been presented to substantiate this. If diosgenin indeed reduces intracellular ATP levels, considering the established relationship between ATP depletion, decreased metabolic activity, and the nature of the dormancy associated with persister cell formation [15], it can be hypothesized that diosgenin treatment may affect persister cell formation.

The dual-mechanism therapeutic strategy of targeting both (p)ppGpp synthesis and membrane potential presents diosgenin as a compelling compound for combating bacterial persistence. By simultaneously modulating intracellular metabolic pathways and membrane-associated signaling mechanisms, diosgenin may effectively prevent the formation of persister cells while preserving non-target bacterial viability. Additionally, this dual-action approach may offer a synergistic effect when combined with conventional antibiotics, potentially enhancing antibiotic efficacy and reducing the likelihood of infection relapse.

Therefore, this study investigated the potential of diosgenin as a dual-action inhibitor of persister cell formation in *S. aureus*. The primary objective is to evaluate whether diosgenin can downregulate the expression of *relP* and *relQ*, thereby inhibiting (p)ppGpp synthesis and preventing the induction of the stringent response. Additionally, the study tested diosgenin’s effects on membrane potential and intracellular ATP levels, assessing whether the metabolic disruption it causes contributes to persister cell suppression under ciprofloxacin, gentamicin, and oxacillin exposure.

The significance of this study lies in its focus on preventing persister cell formation through metabolic modulation rather than merely eradicating persister cells after they have already formed. If successful, our findings may establish diosgenin as a promising adjunctive therapy for chronic bacterial infections in which persister cells contribute to prolonged infection duration and treatment failure, particularly those involving *S. aureus*. Moreover, the identification of a naturally derived, low-toxicity compound capable of dual mechanism action could serve as a foundation for the development of novel anti-persister therapeutics aimed at targeting both metabolic and signaling pathways.

## 2. Results

### 2.1. Impact of Diosgenin on Bacterial Growth

**Figure 1:**
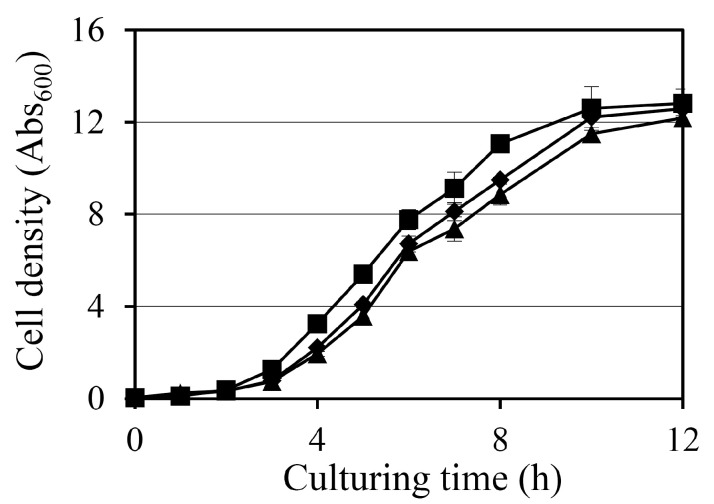
Effect of diosgenin on *Staphylococcus aureus* growth: growth curves of *S. aureus* cultures treated with ethanol (■; control) or 80 µM (◆) or 160 µM (▲) diosgenin over a 12 h period. Bacterial growth was monitored by measuring absorbance at 600 nm (Abs_600_). Data are presented as means ± the standard deviation of three independent experiments.

The effect of diosgenin on the growth of *Staphylococcus aureus* was assessed by monitoring the absorbance at 600 nm (Abs_600_) over a 12 h period in cultures treated with 80 µM and 160 µM diosgenin (Figure 1). In the control group, treated with ethanol only, bacterial cultures exhibited typical exponential growth, reaching an Abs_600_ of 11.1 after 8 h. Treatment with 80 µM diosgenin resulted in a slight but statistically significant reduction in bacterial growth, with the Abs_600_ reaching 9.5 at 8 h. This suggests that 80 µM diosgenin exerts a mild inhibitory effect on bacterial proliferation without substantially arresting growth. A more pronounced inhibitory effect was observed in cultures treated with 160 µM diosgenin, with Abs_600_ values at 8 h reduced to 8.9, representing a further reduction in bacterial growth below that of the 80 µM treatment group. The trend indicates that diosgenin exhibits concentration-dependent suppression of bacterial growth under these conditions. Despite the observed Abs_600_ reductions, the bacterial cultures continued to exhibit growth over the 12 h period at both diosgenin concentrations, suggesting that the inhibitory effects of diosgenin were bacteriostatic rather than bactericidal under the tested conditions.

### 2.2. Effect of Diosgenin on Persister Cell Formation

**Figure 2:**
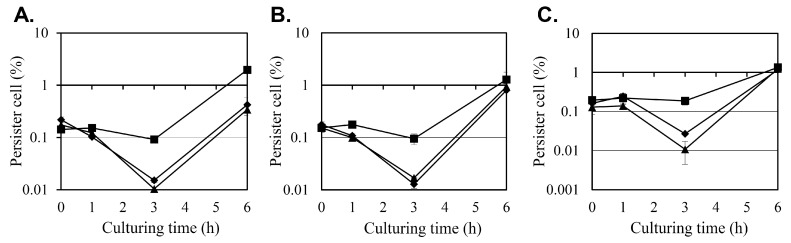
Effects of diosgenin on persister cell survival in *Staphylococcus aureus* under antibiotic stress. The survival of persister cells was assessed in *Staphylococcus aureus* cultures pretreated with ethanol (■; control) or 80 µM (◆) or 160 µM (▲) diosgenin dissolved in ethanol after exposure to oxacillin (**A**), ciprofloxacin (**B**), or gentamicin (**C**) for 24 h, with antibiotic treatments administered immediately (0 h) or 1, 3, or 6 h after diosgenin exposure (*x*-axis). The antibiotics were applied at concentrations of 2.5 mg/L for oxacillin, 20 mg/L for ciprofloxacin, and 80 mg/L for gentamicin, representing 10 × their respective MICs, in the treatments at 0, 1, and 3 h but at concentrations of 25, 200, and 800 mg/L (100 × MIC), respectively, in the treatment at 6 h. The number of viable cells was quantified as colony-forming units (CFU) and is represented as the ratio of CFU after antibiotic exposure to the initial CFU count before antibiotic administration. Data are presented as means ± the standard deviation of three independent experiments.

The effect of diosgenin on *S. aureus* persister cell formation was evaluated under antibiotic stress induced by oxacillin, ciprofloxacin, and gentamicin at 10 × minimum inhibitory concentration (MIC) (Figure 2). Persister cell survival was quantified as colony-forming units (CFU), evaluated as the percentage of surviving cells relative to the initial CFU count, with antibiotics administered 0, 1, 3, or 6 h after the diosgenin pretreatment.

In the control group, pretreated with ethanol instead of diosgenin only, the number of persister cells remained relatively stable when antibiotics were administered during the first 3 h after the ethanol treatment, regardless of the antibiotic used. However, a substantial increase in persister cell formation was observed when antibiotics were administered after 6 h, with persister cell counts increasing 21-, 13-, and 7-fold between the treatments at hours 3 and 6 for oxacillin, ciprofloxacin, and gentamicin, respectively. This surge in persister cell populations coincides with the transition of *S. aureus* cultures into the stationary phase (Figure 1), indicating that persister cell formation may be associated with metabolic downregulation during growth arrest.

Pretreatment with 80 µM and 160 µM diosgenin significantly reduced persister cell formation at the 3 h time point across all antibiotic treatments. Under oxacillin stress, the persister cell fraction in the control group was 0.0919%, but pretreatment with 80 µM and 160 µM diosgenin reduced the persister cell fraction to 0.015% and 0.0104%, representing 83% and 89% reductions, respectively. A similar inhibitory effect was observed under ciprofloxacin treatment. In the control group, the persister cell fraction was 0.095%, while pretreatment with 80 µM diosgenin decreased it to 0.0169%, and pretreatment with 160 µM diosgenin further reduced it to 0.0128%, corresponding to reductions of 82% and 87%, respectively. In cultures treated with gentamicin, persister cell formation was also markedly reduced by diosgenin pretreatment. The control group exhibited a persister cell fraction of 0.187%, which was reduced to 0.0271% and 0.0108% in the 80 µM and 160 µM diosgenin-treated cultures, respectively. These reductions correspond to decreases of 85% and 94%, respectively, indicating that diosgenin effectively suppresses persister cell formation 3 h after it is applied across different antibiotic treatments.

At the 6 h time point, persister cell fractions in the diosgenin-treated groups began to increase, aligning with the trend observed in the control group. This increase coincides with the onset of the stationary phase, during which metabolic dormancy may contribute to persister cell survival. These findings indicate that diosgenin exerts a time- and concentration-dependent inhibitory effect on persister cell formation, particularly before the metabolic downregulation associated with the stationary phase occurs.

### 2.3. Effect of Diosgenin on relP and relQ Expression

**Figure 3:**
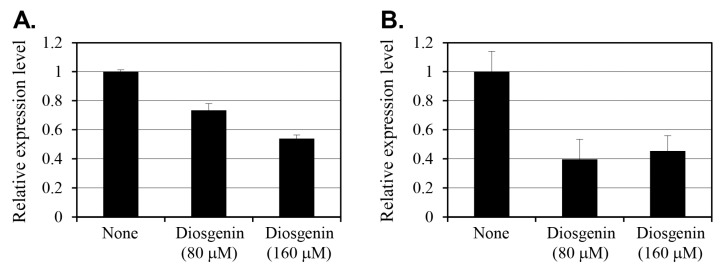
Effect of diosgenin on *relP* (**A**) and *relQ* (**B**) gene expression in *Staphylococcus aureus. Staphylococcus aureus* cells were treated with 80 µM and 160 µM diosgenin, and relative expression was determined using RT-PCR analysis. Gene expression levels were normalized to that of the constitutively expressed 16s rRNA gene and expressed relative to an untreated control (None). Data are presented as means ± the standard deviation of three independent experiments.

The effect of diosgenin on the expression of *relP* and *relQ*, the two key genes involved in (p)ppGpp synthesis, was evaluated using reverse transcription-polymerase chain reaction (RT-PCR) on *S. aureus* cells cultured with 80 µM or 160 µM diosgenin for 3 h (Figure 3). Gene expression levels were normalized to 16s rRNA levels and are expressed as fold changes relative to an untreated control.

In cells treated with 80 µM diosgenin, *relP* expression decreased by 27% compared to that in control group cells. Treatment with 160 µM diosgenin reduced *relP* expression by 46%, indicating a dose-dependent suppression of *relP* transcription in response to diosgenin. Conversely, *relQ* expression exhibited a more pronounced reduction in response to diosgenin treatment. At 80 µM diosgenin, *relQ* expression decreased by 60% relative to the control, representing a substantial reduction. Interestingly, increasing the diosgenin concentration to 160 µM resulted in a slightly lower 55% reduction. This suggests that *relQ* expression was already substantially suppressed at 80 µM, with no further dose-dependent decrease observed at 160 µM.

The differential responses of *relP* and *relQ* to diosgenin treatment indicate that distinct regulatory mechanisms underlie the expression of these (p)ppGpp synthase genes. While *relP* expression demonstrates a clear dose-dependent reduction, diosgenin’s suppressive effect on *relQ* expression appears to reach a threshold at or below 80 µM, beyond which increasing the diosgenin concentration has no effect. Overall, these findings suggest that diosgenin selectively modulates *S. aureus*’ stringent response pathway by targeting *relP* and *relQ* transcription, potentially disrupting (p)ppGpp synthesis and thereby inhibiting persister cell formation.

### 2.4. Intracellular ATP Levels in Response to Diosgenin

**Figure 4:**
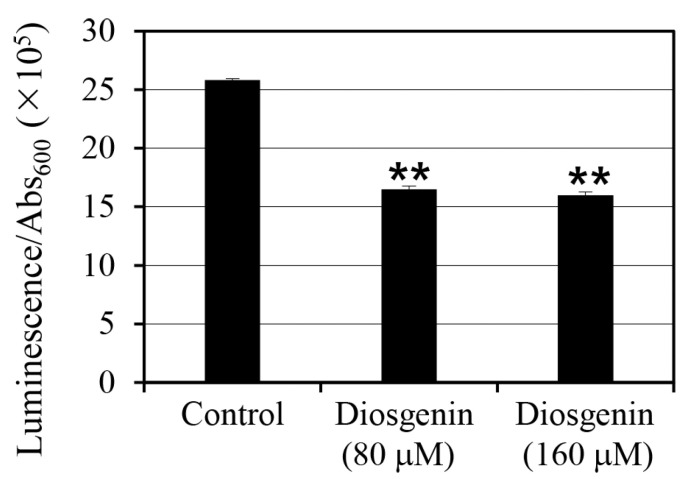
Effect of diosgenin on intracellular ATP levels in *Staphylococcus aureus. Staphylococcus aureus* cells were cultured in 80 µM and 160 µM diosgenin media, and ATP levels were quantified by luminescence and normalized to cell density (Abs_600_) to calculate intracellular ATP per cell. Data are presented as means ± the standard deviation of three independent experiments. Statistically significant differences at the 99% confidence level (*p* < 0.01) between the treatment means and the control mean are indicated by asterisks (“**”).

To assess the effect of diosgenin on intracellular ATP levels in *S. aureus*, ATP was quantified using luminescence assays on cells cultured in 80 µM and 160 µM diosgenin media (Figure 4). ATP levels were normalized to cell density (Abs_600_), accounting for variations in cell growth, to provide a more accurate representation of intracellular ATP content per cell. Treatment with 80 µM diosgenin resulted in a 36% reduction in intracellular ATP levels relative to the control group, indicating a significant decrease in cellular energy reserves (*p* < 0.01). A similar reduction was observed in cultures treated with 160 µM diosgenin, where intracellular ATP levels decreased by 38% compared to the control group (*p* < 0.01). Despite the higher diosgenin concentration, the ATP reduction was similar to that observed at 80 µM, suggesting that the inhibitory effect of diosgenin on ATP synthesis may reach a threshold at or before 80 µM. These reductions in intracellular ATP levels indicate that diosgenin disrupts metabolic processes associated with energy production, potentially contributing to its inhibitory effect on persister cell formation. Since ATP is a critical metabolite required for the survival and stress response of persister cells, the observed ATP depletion may reduce cellular viability under antibiotic stress, further enhancing the anti-persister effects of diosgenin.

### 2.5. Membrane Permeability and Fluidity Analysis

**Figure 5:**
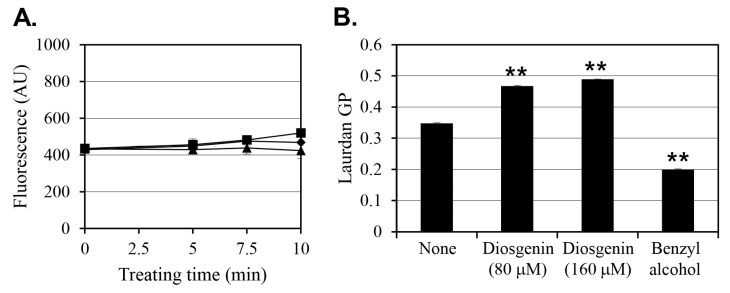
Effect of diosgenin on membrane permeability and fluidity in *Staphylococcus aureus* cells. (**A**) Membrane permeability of *S. aureus* cells treated with ethanol (■; control) or 80 µM (◆) or 160 µM (▲) diosgenin, as measured by fluorescence intensity (arbitrary unit, AU). (**B**) Membrane fluidity of *S. aureus* cultures treated with 80 µM or 160 µM diosgenin and untreated control cultures (none). Benzyl alcohol was used as a positive control for membrane fluidization. Data are presented as means ± the standard deviation of three independent experiments. Statistically significant differences at the 99% confidence level (*p* < 0.01) between the treatment means and the control mean are indicated by asterisks (“**”).

Previous studies have suggested that increasing membrane permeability, enhancing antibiotic uptake, can be an effective strategy for controlling persister cells. To investigate whether diosgenin affects membrane permeability in *S. aureus*, fluorescence assays were conducted using 80 µM and 160 µM diosgenin (Figure 5A). In the control group, baseline membrane permeability values remained relatively constant. Contrary to expectations, treatment with diosgenin at either concentration did not result in any significant change in membrane permeability compared to the control group. Fluorescence intensity values were statistically indistinguishable across all treatments, indicating that diosgenin does not significantly alter membrane permeability under the tested concentrations. These findings align with the observation that diosgenin does not exhibit bactericidal activity against pre-formed persister cells (Supplementary Figure S2).

Next, considering evidence that diosgenin is structurally capable of interacting with membrane lipids, its effect on membrane fluidity was assessed to determine potential impacts on membrane-associated protein activity (Figure 5B). Benzyl alcohol, a known membrane fluidizer, was used as a positive control to validate the assay’s responsiveness. In cells cultured with 80 µM diosgenin, membrane fluidity decreased by 35% relative to the untreated control, and the 160 µM diosgenin treatment decreased membrane fluidity by 41%, indicating that diosgenin induces membrane rigidification in a dose-dependent manner. In contrast, benzyl alcohol increased membrane fluidity by 43%, confirming its expected fluidizing effect.

To further explore whether diosgenin affects membrane potential, fluorescent membrane potential indicators were utilized to assess potential changes under 80 µM and 160 µM diosgenin treatment (Supplementary Figure S1). No significant differences in membrane potential were found between diosgenin-treated and control groups, suggesting that diosgenin does not substantially alter membrane polarization at the tested concentrations.

The observed reductions in membrane fluidity without concurrent increases in membrane permeability or significant changes in membrane potential suggest that diosgenin’s primary mechanism of action may not involve membrane disruption but rather the modulation of membrane-associated signaling. Membrane fluidity is known to influence the activity of embedded proteins and receptors, potentially affecting stress response pathways and persister cell signaling mechanisms. Thus, the decrease in membrane fluidity induced by diosgenin may alter the functional dynamics of membrane-associated proteins, contributing to its inhibitory effect on persister cell formation.

## 3. Discussion

The present study provides new insights into the mechanisms by which diosgenin modulates persister cell formation in *S. aureus*. By systematically analyzing its effects on bacterial growth, persister cell formation, (p)ppGpp synthase gene expression, intracellular ATP levels, and membrane integrity, we have identified multiple pathways through which diosgenin exerts anti-persister activity.

Diosgenin exhibited a dose-dependent inhibitory effect on bacterial growth, with 80 µM and 160 µM treatments reducing cell density by 9% and 12%, respectively (Figure 1). Although the reductions were modest, they were statistically significant and indicate that diosgenin exerts a mild bacteriostatic effect rather than bactericidal action. This observation is consistent with previous reports identifying diosgenin as a metabolic modulator without direct bactericidal properties. The more pronounced effect of diosgenin was observed in its reduction in persister cell populations under antibiotic stress (Figure 2). Pretreatment with 80 µM and 160 µM diosgenin significantly decreased persister cell fractions across oxacillin, ciprofloxacin, and gentamicin treatments, with reductions ranging from 82% to 94% 3 h after diosgenin application. This reduction aligns with a previous study showing that metabolic disruption can effectively inhibit persister cell formation by preventing the metabolic shift to dormancy [27]. Interestingly, we found that persister cell inhibition was most pronounced during a relatively short period after diosgenin exposure (1–3 h), with the effect diminishing after 6 h, as cultures transitioned to the stationary phase. This trend suggests that diosgenin may prevent the initial establishment of persister cells but is less effective once persister cells are fully established, highlighting a temporal limitation in its anti-persister activity.

The stringent response, mediated by (p)ppGpp, plays a pivotal role in persister cell formation by downregulating metabolic activity under stress conditions [28]. In this study, diosgenin treatments significantly reduced the expression of *relP* and *relQ*, the two key synthases involved in (p)ppGpp synthesis (Figure 3). When cultured in the 80 µM diosgenin medium, *relP* expression in *S. aureus* cells decreased by 27%, while the 160 µM diosgenin treatment reduced *relP* expression by 46%. The dose-dependent suppression of *relP* is particularly relevant given its established role in (p)ppGpp synthesis. In contrast, *relQ* expression exhibited a more substantial reduction in 80 µM diosgenin-treated cells (60%), with no further reduction when the concentration increased to 160 µM (55%). This suggests that *relQ* expression is more sensitive to diosgenin and that a suppression threshold exists at or below 80 µM, beyond which increasing the diosgenin concentration does not significantly enhance inhibition. Such a plateau in *relQ* suppression may indicate that other regulatory factors limit the extent of diosgenin-induced inhibition at higher concentrations. The plateau in *relQ* suppression at 80 µM diosgenin, despite further increases in diosgenin concentration, may result from several regulatory dynamics. First, *relQ* transcription is known to be responsive to specific environmental stress signals and may be governed by promoter elements less susceptible to repression by membrane or metabolic perturbations beyond a threshold level. Second, feedback regulation through (p)ppGpp levels or sigma factor activity could stabilize *relQ* expression once an initial suppression occurs. Notably, Horvatek et al. [14] reported that *relQ* is inducible under specific conditions of amino acid starvation and oxidative stress, which may buffer it against further downregulation in the presence of sustained metabolic inhibition. Lastly, functional overlap between *relP* and *relQ* might allow the cell to maintain a basal (p)ppGpp synthesis capability by preserving minimal *relQ* expression, particularly in the face of external modulators such as diosgenin. These hypotheses underscore the complexity of stringent response regulation and warrant further transcriptional profiling studies under varying stress and inhibitor conditions. Additionally, the differential response of *relP* and *relQ* to diosgenin treatment suggests that distinct regulatory mechanisms govern the expression of these synthases, as was suggested in a previous study [14].

Another major finding in this study was that diosgenin treatment significantly reduces intracellular ATP levels in *S. aureus* (Figure 4). Treatment with 80 µM diosgenin reduced intracellular ATP levels by 36%, while 160 µM diosgenin decreased ATP levels by 38%. These reductions suggest that diosgenin disrupts ATP synthesis pathways, potentially limiting the energy resources required for persister cell formation and maintenance. The similar extent of ATP reduction at both diosgenin concentrations indicates that diosgenin’s inhibitory effect may reach a plateau at or before 80 µM. It should be noted that the results in this study were calculated based on the ATP concentration in the lysate solution obtained after cell disruption, not the intracellular ATP concentration. To estimate ATP concentration on a per-cell basis, the measured ATP concentration was divided by the cell concentration. Given the critical role of ATP in supporting the dormancy and resuscitation of persister cells, the observed depletion may significantly impact persister cell survival. Moreover, when coupled with the decreased expression of *relP* and *relQ*, these findings suggest that diosgenin disrupts the metabolic framework that underpins stringent response signaling.

Previous studies have proposed that disrupting the membrane integrity of persister cells can sensitize them to antibiotics by enhancing intracellular drug uptake [27]. Diosgenin did not significantly alter membrane permeability at either 80 or 160 µM (Figure 5A), indicating that its anti-persister activity is not mediated through membrane disruption. However, diosgenin markedly decreased membrane fluidity in a dose-dependent manner, with the 80 µM treatment reducing fluidity by 35% and the 160 µM treatment reducing it by 41% (Figure 5B). The extent of membrane rigidification at 160 µM was comparable, though in the opposite direction, to the increase in membrane fluidity induced by benzyl alcohol, a known membrane fluidizer [29]. These reductions in membrane fluidity suggest that diosgenin interacts with membrane lipids, potentially altering membrane-associated signaling pathways involved in persister cell regulation. It is interesting that, despite these changes, no significant alterations in membrane potential were detected at any concentration (Supplementary Figure S1). This finding indicates that under the tested conditions, diosgenin affects membrane dynamics but does not disrupt ion gradients or membrane polarization.

Taken together, our findings in this study suggest that diosgenin reduces persister cell formation through multiple mechanisms involving metabolic suppression, stringent response inhibition, and membrane modulation: The substantial reductions in *relP* and *relQ* expression, coupled with ATP depletion, indicate that diosgenin primarily targets the metabolic pathways essential for persister cell survival. The observed decrease in membrane fluidity, without concomitant increases in membrane permeability or significant changes in membrane potential, suggests that diosgenin may selectively affect membrane-associated signaling proteins involved in stress response pathways. This may contribute to its anti-persister activity by altering the functional dynamics of membrane-bound receptors or enzymes. However, the temporal limitation of diosgenin’s anti-persister effects, specifically its reduced efficacy 6 h after exposure, suggests that its inhibitory activity may be most effective during the early phase. This observation highlights the need for further studies to determine whether combining diosgenin with other metabolic inhibitors or membrane-targeting agents can prolong its anti-persister effects for extended periods. Although our findings were derived from in vitro experiments, they provide a mechanistic foundation for future in vivo evaluations. Assessing diosgenin’s bioavailability, toxicity, and therapeutic efficacy in animal models of persistent infection will be essential to determine its translational potential.

Several factors may contribute to the diminished efficacy of diosgenin after 6 h of exposure. First, diosgenin may undergo chemical degradation or reduced bioavailability in the culture medium over time, which could weaken its biological activity. Second, bacterial adaptation mechanisms such as the upregulation of efflux pumps or changes in membrane composition may reduce cellular sensitivity to diosgenin during prolonged exposure. Third, once *S. aureus* enters the stationary phase, the established persister cells may possess robust metabolic and structural defenses that render them intrinsically less susceptible to interventions targeting earlier phases of dormancy initiation. These possibilities merit further investigation and underscore the need for kinetic studies of diosgenin stability and bacterial adaptive responses over extended treatment periods.

Despite this study’s promising findings, it has limitations that warrant further investigation. First, all our experiments were conducted in vitro, and the potential effects of diosgenin in vivo should be explored. Additionally, though we identified reductions in (p)ppGpp synthase gene expression and ATP levels, the precise molecular interactions between diosgenin and metabolic enzymes remain unclear. Future studies should focus on elucidating the molecular targets of diosgenin within the stringent response and metabolic pathways, as well as assessing its effects in infection models. Moreover, exploring potential synergies between diosgenin and conventional antibiotics may provide valuable insights into its clinical applicability as an anti-persister agent in chronic and recurrent infections.

## 4. Materials and Methods

### 4.1. Bacterial Strains and Culture Conditions

*Staphylococcus aureus* strain ATCC 6538 was obtained from the Korean Collection for Type Cultures (Jeongeup, Republic of Korea) and stored at −80 °C in Tryptic Soy Broth (TSB; catalog no. 211825, Becton Dickinson Korea Co., Ltd., Seoul, Republic of Korea) containing 25% glycerol (catalog no. 4066-4400, Daejung Chemicals & Metals Co., Ltd., Siheung, Republic of Korea). Prior to each experiment, single colonies were streaked onto TSB agar plates containing 1.5% agar (SKU 214010, Becton Dickinson Korea Co., Ltd.) and incubated at 37 °C for 24 h.

To prepare overnight cultures, a single colony was inoculated into 5 mL of TSB and incubated at 37 °C with shaking at 250 rpm for 24 h. Subsequently, the bacterial suspension was diluted with fresh TSB to an Abs_600_ of 0.05 to serve as the starting inoculum for all experiments.

The MIC for *S. aureus* ATCC 6538 was determined in-house using the broth microdilution method according to CLSI guidelines. The MIC values were as follows: 0.25 mg/L for oxacillin, 2.0 mg/L for ciprofloxacin, and 8.0 mg/L for gentamicin.

The strain *S. aureus* ATCC 6538 was chosen due to its widespread use as a reference model in antimicrobial and persistence studies, offering a stable and well-documented genetic background that supports reproducible evaluations of stress responses, membrane dynamics, and antibiotic tolerance.

### 4.2. Diosgenin and Antibiotic Preparation

Diosgenin (catalog no. sc-205652, Santa Cruz Biotechnology Inc., Dallas, TX, USA) was dissolved in 99.9% ethanol (catalog no. 000E0243, Samchun Chemical Co., Ltd., Seoul, Republic of Korea) to prepare stock solutions, which were stored at −20 °C until further use. Prior to each experiment, the diosgenin stock solution was diluted with TSB to the desired working concentration. The antibiotics ciprofloxacin hydrochloride monohydrate (catalog no. C2227, Tokyo Chemical Industry Co., Ltd., Tokyo, Japan), oxacillin sodium salt (catalog no. sc-224180B, Santa Cruz Biotechnology Inc.), and gentamicin sulfate (catalog no. G0383, Tokyo Chemical Industry Co., Ltd.) were dissolved in sterile distilled water before use. All antibiotic stock solutions were filtered through a 0.22 µm membrane filter and stored at −20 °C until use.

### 4.3. Growth Curve Analysis

The effects of diosgenin on bacterial growth were evaluated using a standard growth curve analysis. Cultures were prepared as previously described and adjusted to an Abs_600_ of 0.05 in TSB. Diosgenin was added to the cultures to final concentrations of 80 µM and 160 µM, and control cultures were treated with ethanol. The cultures were incubated at 37 °C with shaking at 250 rpm, and the Abs_600_ was measured every hour for the first 8 h and at hours 10 and 12 using a Synergy™ LX Multi-Mode Reader (BioTek Instruments Korea Ltd., Seoul, Republic of Korea). Growth curves were constructed by plotting the measured Abs_600_ values against time to assess the impact of diosgenin on bacterial growth.

### 4.4. Persister Cell Induction and Diosgenin Treatment

*Staphylococcus aureus* cultures were prepared by inoculating 5 mL of TSB with a single colony and then incubating the inoculated culture at 37 °C for 24 h. For the control group, 0.25 mL of 99.9% ethanol was added to 4.5 mL of TSB, while for the experimental groups, diosgenin dissolved in 0.25 mL of 99.9% ethanol was added to 4.5 mL of TSB to concentrations of 80 µM or 160 µM. The pre-cultured bacterial strains were then inoculated at an initial Abs_600_ of 0.05, and the cultures were incubated at 37 °C with shaking at 250 rpm. To induce persister cell formation, one of three antibiotics (oxacillin, ciprofloxacin, or gentamicin) was administered at one of four time points: immediately (0 h) or 1, 3, or 6 h after inoculation. To mimic standard persister assay conditions and ensure complete elimination of metabolically active cells, antibiotics were administered at 10 × MIC (2.5 mg/L oxacillin, 20 mg/L ciprofloxacin, and 80 mg/L gentamicin) at 0, 1, and 3 h post-inoculation. At the 6 h time point—corresponding to entry into the stationary phase—antibiotics were added at 100 × MIC (25 mg/L oxacillin, 200 mg/L ciprofloxacin, and 800 mg/L gentamicin) to account for increased tolerance in late-phase cells. These high concentrations are commonly used in persister studies to selectively kill growing cells and assess the survival of dormant, drug-tolerant persister cells. The antibiotics do not promote further growth under these conditions; instead, they create a selective pressure that allows quantification of surviving persister cells via CFU counts after antibiotic exposure. Following antibiotic administration, cultures were incubated at 37 °C with shaking at 250 rpm for 24 h. After incubation, the cultures were serially diluted using saline and spread onto TSB agar plates. The experiment was conducted in triplicate, and the results were evaluated as the ratio of CFU before antibiotic treatment to CFU after 24 h of antibiotic exposure.

### 4.5. RNA Extraction and RT-PCR Analysis

Total RNA was extracted from metabolically active, mid-log phase cultures treated with diosgenin for 3 h using a Tissuelyzer LT (catalog no. 85600, Qiagen Korea Co., Seoul, Republic of Korea) and the AccuPrep Bacterial RNA Extraction Kit (catalog no. K-3142, Bioneer Co., Daejeon, Republic of Korea). These samples represent growing cells rather than persister cells to assess early gene expression responses prior to dormancy induction. cDNA was synthesized using the TOPreal™ Probe RT-qPCR Kit (catalog no. RT430S, Enzynomics Co., Ltd., Daejeon, Republic of Korea) and a QuantStudio 5 RT-PCR system (catalog no. A34322, Thermo Fisher Scientific Korea Ltd., Seoul, Republic of Korea).

The RT-PCRs were performed using the primers *relP* forward (5′-ACTTGGGTTGGTGCATACGT-3′), *relP* reverse (5′-GATTTCGAGCGGGTCTCCAT-3′), *relQ* forward (5′-AAGATTGGCCGAAACCAGGT-3′), *relQ* reverse (5′-CGCTTCAGGTCCTACAACGA-3′), 16S rRNA forward (5′-TCCGGAATTATTGGGCGTAA-3′), and 16S rRNA reverse (5′-CCACTTTCCTCTTCTGCACTCA-3′). The thermocycling conditions consisted of 50 °C for 30 min and then 95 °C for 10 min, followed by 35 cycles of 95 °C for 15 s, 60 °C for 30 s, and 72 °C for 30 s. To confirm the specificity of the PCR products, melting curve analyses were performed from 60 °C to 95 °C. The entire gene expression experiment was conducted in triplicate.

### 4.6. Intracellular ATP Analysis

Intracellular ATP levels were quantified using an ATP Determination Kit (catalog no. A22066, Thermo Fisher Scientific Korea Ltd.). Bacterial cultures treated with diosgenin were centrifuged at 6800× *g* for 5 min and resuspended in phosphate-buffered saline (PBS). Then, ATP levels were measured based on luminescence using a Synergy™ LX Multi-Mode Reader, and the measured luminescence values were divided by cell density (Abs_600_) measurements to calculate per-cell ATP concentrations.

### 4.7. Membrane Permeability Assay

The effects of diosgenin on membrane permeability were assessed using SYTOX™ Green Nucleic Acid Stain (catalog no. S7020, Thermo Fisher Scientific Korea Ltd., Seoul, Republic of Korea). Mid-log phase cultures were treated with diosgenin at 80 µM and 160 µM concentrations for 3 h. The cultures were then centrifuged at 6800× *g* for 5 min and washed twice with sterile saline. After washing, the bacterial pellets were resuspended in 1 mL of saline, and SYTOX™ Green Nucleic Acid Stain was added to a final concentration of 0.5 µM. The samples were incubated at room temperature in the dark for 10 min to allow for dye uptake. Fluorescence was then measured at an excitation wavelength of 485 nm and an emission wavelength of 528 nm using a Synergy™ LX Multi-Mode Reader. Positive control samples were treated with 70% ethanol to induce membrane permeabilization.

### 4.8. Membrane Fluidity Analysis

Membrane fluidity was assessed using Laurdan (catalog no. D250, Thermo Fisher Scientific Korea Ltd.). Three-hour cultures of *S. aureus* were treated with 10 µM of Laurdan and incubated for 10 min. The cells were then washed three times with PBS using centrifugation at 8500× *g* for 5 min and resuspended in PBS, with the original volume. Following 80 µM or 160 µM diosgenin treatment or benzyl alcohol treatment (positive control), 200 µL of sample was dispensed into the wells of a black 96-well plate and incubated at room temperature for 1 h. Fluorescence was subsequently measured using a Varioskan™ LUX Multimode Microplate Reader (catalog no. VLBL00D0, Thermo Fisher Scientific Korea Ltd.) with excitation at 350 nm and emissions recorded at 435 and 490 nm. Laurdan GP values were calculated as (*I*_435_ − *I*_490_)/(*I*_435_ + *I*_490_). The experiment was conducted in triplicate.

### 4.9. Statistical Analysis

Data were analyzed using GraphPad Prism (version 9.0). Statistical significance was assessed using one-way ANOVAs followed by comparisons between control and treatment groups using Student’s *t*-tests. Data are presented as means ± standard deviations, and *p*-values < 0.05 were considered statistically significant.

## 5. Conclusions

This study demonstrates that diosgenin effectively reduces persister cell formation in *Staphylococcus aureus* through mechanisms involving metabolic suppression and membrane modulation. Diosgenin treatments significantly decreased persister cell survival under antibiotic stress, particularly during a relatively short period after exposure. An inhibition of *relP* and *relQ* gene expression and an associated reduction in intracellular ATP levels suggest that diosgenin disrupts key metabolic pathways that are essential for persister cell maintenance. Moreover, diosgenin selectively altered membrane fluidity without affecting membrane permeability or membrane potential, indicating a targeted effect on membrane-associated signaling rather than membrane integrity, which may further contribute to diosgenin’s inhibitory effects on persister cells by influencing the dynamics of membrane proteins involved in stress response pathways. While our findings support diosgenin’s potential as an anti-persister agent, its efficacy appears to diminish as cultures enter the stationary phase, highlighting the need for further studies assessing its long-term effectiveness and potential synergistic effects with conventional antibiotics. Future research should focus on in vivo validation and exploring combination therapies to optimize diosgenin’s anti-persister activity. In summary, diosgenin demonstrates a dual mechanism of action against *S. aureus* persister cells by targeting both metabolic and membrane-associated pathways, making it a promising compound for use in strategies to mitigate chronic and recurrent bacterial infections.

## Data Availability

The original contributions presented in this study are included in the article/Supplementary Material. Further inquiries can be directed to the corresponding author.

## Supplementary Materials

The following supporting information can be downloaded at https://www.mdpi.com/article/10.3390/ijms26136335/s1.

## Abbreviations

The following abbreviations are used in this manuscript:

MIC	minimum inhibitory concentration
CFU	colony-forming unit
RT-PCR	reverse transcription-polymerase chain reaction
TSB	Tryptic Soy Broth
