# Supplementary material for: Inhibition of (p)ppGpp Synthesis and Membrane Fluidity Modulation by Diosgenin: A Strategy to Suppress Staphylococcus aureus Persister Cells
Source: Int J Mol Sci. 2025 Jun 30;26(13):6335. doi: 10.3390/ijms26136335 (PMC12249486; doi:10.3390/ijms26136335)
Supplement: Supplementary file 1 [file ijms-26-06335-s001.zip › ijms-3714665-supplementary.pdf]

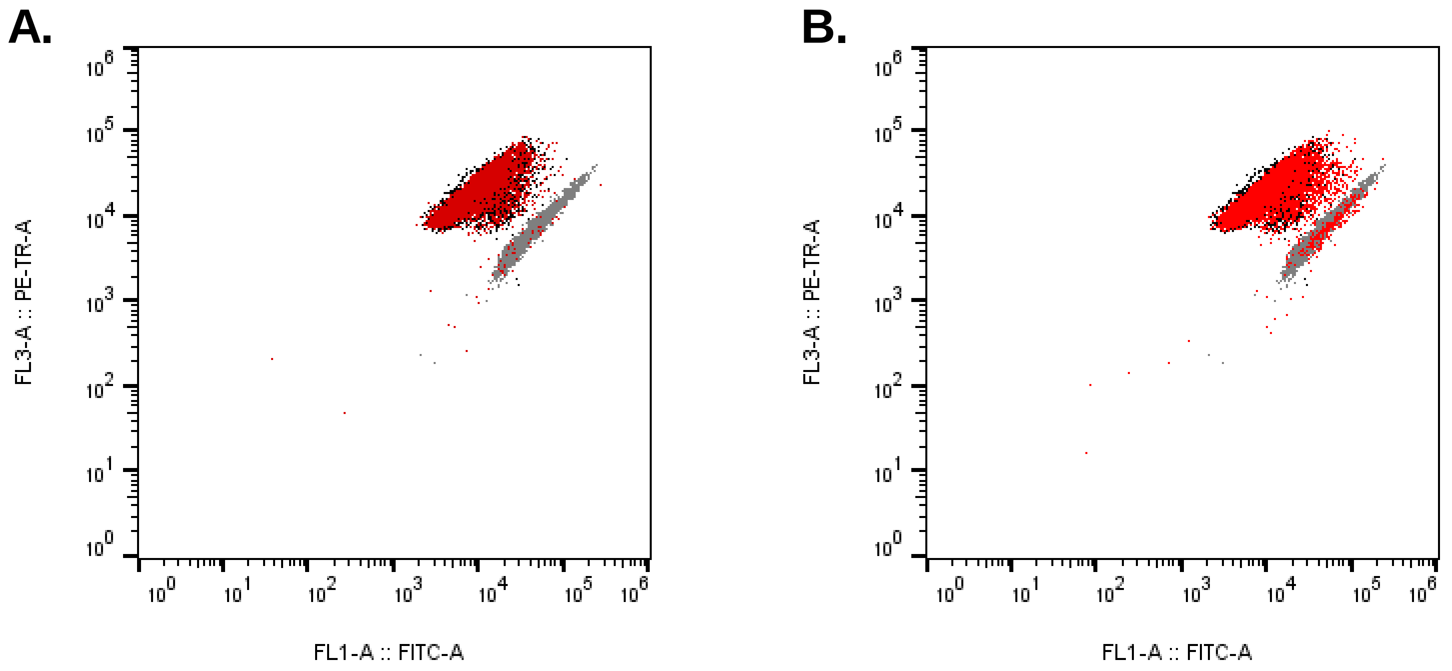

Supplementary Figure S1. Effect of Diosgenin on the Membrane Potential of *Staphylococcus aureus* cells

(A) Membrane potential analysis in *Staphylococcus aureus* treated with 80 µM diosgenin.

(B) Membrane potential analysis in *Staphylococcus aureus* treated with 160 µM diosgenin.

Membrane potential was assessed using fluorescent indicators, with untreated control cultures serving as the baseline reference. Data are presented as the mean ± standard deviation of three independent experiments. No significant changes in membrane potential were observed in either treatment group compared to the control.

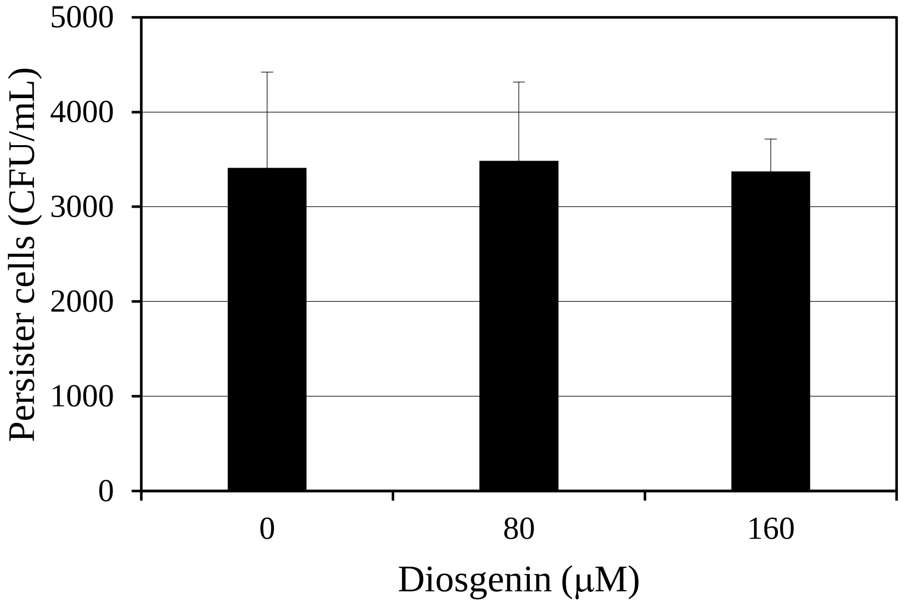

Supplementary Figure S2. Effect of Diosgenin on Viability of Pre-Formed *Staphylococcus aureus* Persister Cells. Persister cells were induced by treating *S. aureus* cultures with 2.5 mg/L oxacillin for 24 h. Surviving persister cells were collected, washed, and resuspended in fresh tryptic soy broth (TSB) containing either 80 µM or 160 µM diosgenin, or ethanol as a control. After a 24-hour incubation period, colony-forming units (CFUs) were quantified. Diosgenin treatment did not result in a significant reduction in CFU counts compared to the control group in 6 h, indicating that diosgenin does not exhibit bactericidal activity against pre-formed persister cells. Data represent the mean ± standard deviation of three independent experiments. Statistical analysis was performed using one-way ANOVA with post hoc t-tests; no statistically significant differences were observed ($p > 0.05$).
